# Supplementary figures and images for: Transboundary movements of foot-and-mouth disease from India to Sri Lanka: A common pattern is shared by serotypes O and C
Source: PLoS One. 2019 Dec 31;14(12):e0227126. doi: 10.1371/journal.pone.0227126 (PMC6938362; doi:10.1371/journal.pone.0227126)

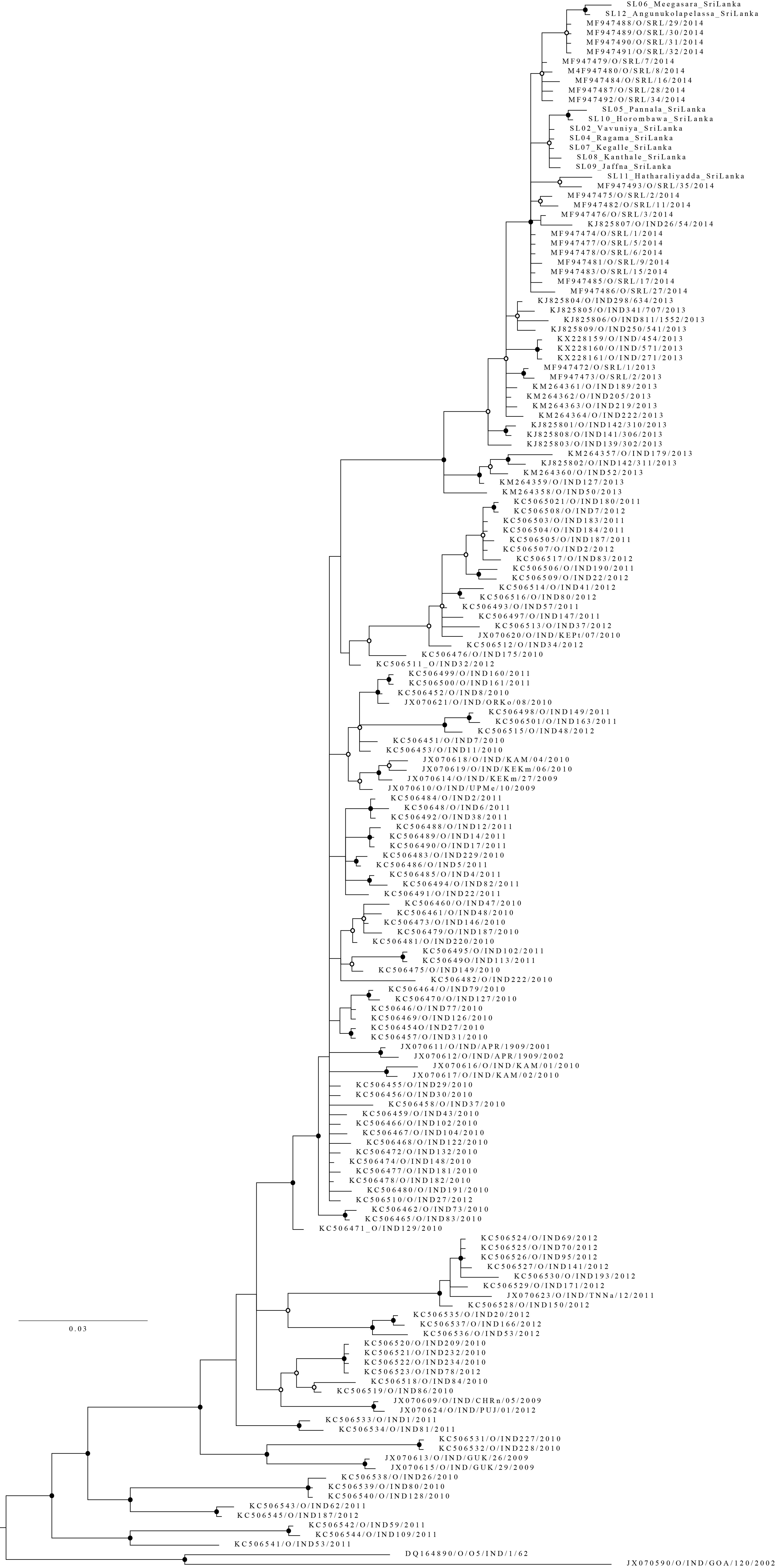

Supplement: S1 Fig — The Black dots indicate the nodes with PP>90 and bs>70. The grey dots indicate the node with PP>90. The white dots with black outlines indicate the nodes with bs<70. (TIF) [file pone.0227126.s001.tif]
