# Supplementary material for: Transboundary movements of foot-and-mouth disease from India to Sri Lanka: A common pattern is shared by serotypes O and C
Source: PLoS One. 2019 Dec 31;14(12):e0227126. doi: 10.1371/journal.pone.0227126 (PMC6938362; doi:10.1371/journal.pone.0227126)
Supplement: Attachment — Submitted filename: Response to Reviewers.docx [file pone.0227126.s006.docx]

**Prof. S.D.S.S. Sooriyapathirana**,

Corresponding Author,

Head,

Dept. of Molecular Biology and Biotechnology,

November 11, 2019

Editor,

PLOS ONE.

Dear Editor,

Rebuttal Letter Containing Comments and Answers for the manuscript titled “**Transboundary movements of foot-and-mouth disease from India to Sri Lanka: a common pattern is shared by serotypes O and C**”

I wish to pay my sincere thanks for reviewing and taking necessary actions to review the earlier submitted manuscript titled “Transboundary movements of foot-and-mouth disease from India to Sri Lanka: a common pattern is shared by serotypes O and C”. I appreciate your valuable effort in this regard to point out areas to improve and strengths in our study which are helpful in improving the quality of the article. This article is of immense importance to the betterment of the Sri Lankan cattle industry. Therefore, with your valuable suggestions, we wish to provide convey our research findings to the FMD research community.

Herewith I am sending the details of actions, answers and comments for each query indicated by the reviewers/editor.

**Comments to the Author**

1. Is the manuscript technically sound, and do the data support the conclusions?

The manuscript must describe a technically sound piece of scientific research with data that supports the conclusions. Experiments must have been conducted rigorously, with appropriate controls, replication, and sample sizes. The conclusions must be drawn appropriately based on the data presented.

Reviewer #1: Partly

**Answer:** The suggested changes were included in revised form of the article.

2. Has the statistical analysis been performed appropriately and rigorously?

Reviewer #1: Yes

**Answer:** Thank you for the comment.

3. Have the authors made all data underlying the findings in their manuscript fully available?

medians and variance measures should be available. If there are restrictions on publicly sharing data—e.g. participant privacy or use of data from a third party—those must be specified.

Reviewer #1: Yes

**Answer:** Thank you for the comment.

4. Is the manuscript presented in an intelligible fashion and written in standard English?

PLOS ONE does not copyedit accepted manuscripts, so the language in submitted articles must be clear, correct, and unambiguous. Any typographical or grammatical errors should be corrected at revision, so please note any specific errors here.

Reviewer #1: No

**Answer:** The article was edited to correct the language mistakes.

**Review Comments to the Author**

**Reviewer #1**

**Comment 1:** The data present in this study seems to be interesting in knowing the movement of FMD from the Indian subcontinent to Sri Lanka. Authors extended their study to understanding of genetic variability associated with FMDV VP 1 region to emphasize the effects of this virus on cattle and its impact on associated product movement into Sri Lanka. During his study authors are mainly focused on retrieving literature data on FMDV and combined several new sequences of serotype C. Majority of those sequences are included Sri Lankan and Indian virus sequences. To infer thee evolutionary and transboundary aspects they used few major software and applications, while the model-based approach is used to detect possible recombination.

**Answer:** Thanks for identifying the importance and strengths of this work.

**Comment 2:** However, some issues related to the article should be solved. Present study authors indicated that they have sequenced viral samples from serotype C. However, they haven’t justified why they used only serotype C instead of using both serotypes O and C.

**Answer:**  Thank you very much for your comment. I believe a clarification is needed for this question. We wanted to cover FMDV incursions associated with both serotype O and serotype C since those are the only reported serotypes to carry out an FMD outbreak situation in Sri Lanka. (Introduction: Lines 121-122, 149 -150). The sequence data for FMDV Serotype O viral samples collected in Sri Lanka were publicly available and included in the analysis (Materials and methods: lines 180 - 181). Although FMDV epidemic situations are reported for Serotype C, the sequence data were not publicly available. Thus, we collected and sequenced the Serotype C data, in order to collectively analyze the viral phylodynamic for each incursion, as we wished to present a completed and more robust scientific story.

**Comment 3:** Describing of the sample selection procedure is unclear. Authors are stated that they have used already collected viral samples. But they were not clearly stated how the present study sample is selected. Original sample number and present study cohort selection should be clearly described in the method section.

**Answer:** Thanks for identifying this. We have added “**We sequenced the viral samples that were collected during the outbreak situations caused by FMDV serotype C in Sri Lanka, India and, a few associated countries**”.

**Comment 4:** In the present study, authors are newly generated only 27 sequences of serotype C. Hence a clear understand of the sample numbers can justify the present study.

**Answer:** We used sequences of all the relevant FMDV serotype C isolates submitted WRLFMD by the sample collectors. The number of samples for serotype C was turned out be 27. There were no other relevant sequences of serotype C available to have a larger sample size.

**Comment 5:** Authors need to expand the description of the structure & phylogenetic significance of VP1 region since that is the major interest of their study.

**Answer:** Thanks for the suggestion. However, I believe the **Paragraph 2** **of the Introduction** justifies the usage of VP1 genomic region and its importance in FMDV phylogenetics.

**Comment 6:** It will be informative that the authors can include the length of the PCR amplicons and the sequences they used to present study. Shorter the sequence length reduced the precision of the analysis.

**Answer:** Thank for pointing this out and making the valuable suggestion. We have remodified the S1 Table including the sequence-lengths.

**Comment 7:** It is understandable if the authors may clearly indicate the number of sequences belongs to each dataset that they used to detect possible recombination events (From line #’s 221 to 224).

**Answer:** Thank you for valuable suggestion. The text in the particular section is corrected accordingly as given below.

“i) serotype C sequences from India and Sri Lanka **(n= 15)**; ii) serotype C sequences from Sri Lanka and sequences of O/ME-SA/Srl-97 **(n = 24)** ; iii) serotype C sequences from Sri Lanka and sequences of O/ME-SA/Ind-2001d from Sri Lanka **(n = 22)**; and iv) O/ME-SA/Ind-2001d sequences from Sri Lanka and India **(n = 150)**”.

**Comment 8:** Some of the references listed in the reference section are not aligned with the journal recommended “Vancouver” reference style.

**Answer:** Thanks for the comment and the references have been double checked to meet the journal style.

**Minor issues**

**Comment:** Reference style in some points of the introduction should be revised and corrected.

Eg: Line # 106: Reference list should be rearranged.

**Answer:** Thanks, corrected accordingly.

**Comment:** Improving written language style is recommended since some language parts of the article are unclear.

**Answer:** Thank you for the suggestion. The relevant changes have been made.

Thank you very much for your consideration and I look forward to hearing back from you with the next step of the publication process.

Thanking you!

Sincerely,

…………………………………..

**Prof. S.D.S.S. Sooriyapathirana** (PhD, Michigan State, USA)

Email: sunethss09@gmail.com

Tel: +94 774464293
