# Supplementary material for: Transboundary movements of foot-and-mouth disease from India to Sri Lanka: A common pattern is shared by serotypes O and C
Source: PLoS One. 2019 Dec 31;14(12):e0227126. doi: 10.1371/journal.pone.0227126 (PMC6938362; doi:10.1371/journal.pone.0227126)
Supplement: Attachment — Submitted filename: Response to Reviewers.docx [file pone.0227126.s007.docx]

**Prof. S.D.S.S. Sooriyapathirana**

Corresponding Author

December 6, 2019

Editor

PLoS ONE

Dear Editor,

**Submission of the Revised Version of PONE-D-19-15471R1: Transboundary movements of foot-and-mouth disease from India to Sri Lanka: a common pattern is shared by serotypes O and C**

We wish to pay our sincere thanks for reviewing the manuscript. Herewith we explain the answers and activities for the queries made by Editor and Reviewer.

**Editor’s Comments (EC):**

**EC1:** Thank you for submitting your manuscript to PLOS ONE. After careful consideration, we feel that it has merit but does not fully meet PLOS ONE’s publication criteria as it currently stands. Therefore, we invite you to submit a revised version of the manuscript that addresses the points raised during the review process.

**Answer:** Thank you very much for the revisions and herewith we submit the revised version.

**EC2:** Please address the few minor comments listed below.

Please consider the following suggestions
1) Line # 157: Please indicates what are the "Nearby" Countries

**Answer:** The ‘Nearby’ countries were included separately in the sentence.

2) Material and Method section: Better rephrase with Passive voice

**Answer:** The Materials and Methods section was rephrased to passive voice.

3)Line # 182: Indicate which type of data set that you used

**Answer:** VP1 sequence datasets were used. The sentence was amended to make it clear.

4)Line # 's 223- 226: Better indicate the individual sequence numbers for each country/Category

**Answer:** We appreciate this comment. The no. of sequences for each country can be easily taken from S1 Table. If we prepare a summary, it would be like the Table given below. Therefore, under the editors permission we wish to keep the current form and guide the readers to S1 Table for sampling details.

| **Country** | **No. of sequences** |
| --- | --- |
| Argentina | 12 |
| Austria | 1 |
| Bangladesh | 2 |
| Belgium | 3 |
| Bhutan | 2 |
| Brazil | 6 |
| Denmark | 1 |
| Ethiopia | 2 |
| France | 4 |
| Germany | 3 |
| Greece | 1 |
| Hungary | 1 |
| India | 108 |
| Italy | 1 |
| Kenya | 3 |
| Kuwait | 1 |
| Nepal | 5 |
| Philipppines | 1 |
| Portugal | 1 |
| Saudi Arabia | 2 |
| Spain | 2 |
| Sri Lanka (Ceylon) | 28 |
| Switzerland | 2 |
| Uganda | 1 |
| United Kingdom | 2 |
| Uruguay | 1 |
| Tajikistan (USSR) | 1 |

5)Line #: Duplication of “Viral”

Answer: We guess the line # has to be 242. The sentence was modified to accommodate the correction.

**Reviewers' comments:**

1. If the authors have adequately addressed your comments raised in a previous round of review and you feel that this manuscript is now acceptable for publication, you may indicate that here to bypass the “Comments to the Author” section, enter your conflict of interest statement in the “Confidential to Editor” section, and submit your "Accept" recommendation.

Reviewer #1: All comments have been addressed
**Answer:** Thank you!

2. Is the manuscript technically sound, and do the data support the conclusions?
The manuscript must describe a technically sound piece of scientific research with data that supports the conclusions. Experiments must have been conducted rigorously, with appropriate controls, replication, and sample sizes. The conclusions must be drawn appropriately based on the data presented.

Reviewer #1: Yes

**Answer:** Thank you!

3. Has the statistical analysis been performed appropriately and rigorously?

Reviewer #1: Yes

**Answer:** Thank you!

4. Have the authors made all data underlying the findings in their manuscript fully available?

The [PLOS Data policy](http://www.plosone.org/static/policies.action#sharing) requires authors to make all data underlying the findings described in their manuscript fully available without restriction, with rare exception (please refer to the Data Availability Statement in the manuscript PDF file). The data should be provided as part of the manuscript or its supporting information, or deposited to a public repository. For example, in addition to summary statistics, the data points behind means, medians and variance measures should be available. If there are restrictions on publicly sharing data—e.g. participant privacy or use of data from a third party—those must be specified.

Reviewer #1: Yes

**Answer:** Thank you!

5. Is the manuscript presented in an intelligible fashion and written in standard English?
PLOS ONE does not copyedit accepted manuscripts, so the language in submitted articles must be clear, correct, and unambiguous. Any typographical or grammatical errors should be corrected at revision, so please note any specific errors here.

Reviewer #1: No

**Answer:** The necessary corrections were made, and the Materials and Methods section was changed to passive voice.

6. Review Comments to the Author
Please use the space provided to explain your answers to the questions above. You may also include additional comments for the author, including concerns about dual publication, research ethics, or publication ethics. (Please upload your review as an attachment if it exceeds 20,000 characters)

Reviewer #1: Please consider the following suggestions
1) Line # 157: Please indicates what are the "Nearby" Countries
2) Material and Method section: Better rephrase with Passive voice
3)Line # 182:  Indicate which type of data set that you used
4)Line # 's 223- 226:  Better indicate the individual sequence numbers for each country/Category
5)Line #: Duplication of "Viral"

**Answer:** All these suggestions were addressed before.

7. PLOS authors have the option to publish the peer review history of their article ([what does this mean?](https://journals.plos.org/plosone/s/editorial-and-peer-review-process#loc-peer-review-history)). If published, this will include your full peer review and any attached files.

If you choose “no”, your identity will remain anonymous but your review may still be made public.

**Do you want your identity to be public for this peer review?** For information about this choice, including consent withdrawal, please see our [Privacy Policy](https://www.plos.org/privacy-policy).

Reviewer #1: Yes: Dr Ruwandi Ranasinghe

**Answer:** Thank you! We would also prefer to publish peer review history according to the journal guidelines.

Again we wish to thank the Editor, Reviewer and PLoS ONE staff for your excellent service. I look forward to hearing back from you with the next step of publication.

Thank you!

Sincerely

Prof. S.D.S.S. Sooriyapathirana

**Corresponding Author**
